# Supplementary material for: MetaRibo-Seq measures translation in microbiomes
Source: Nat Commun. 2020 Jun 29;11:3268. doi: 10.1038/s41467-020-17081-z (PMC7324362; doi:10.1038/s41467-020-17081-z)
Supplement: Supplementary file 10 — Supplementary Data 7 [file 41467_2020_17081_MOESM10_ESM.zip › File2/Confidence_High_Taxonomy/38945_out.krona.html]

Javascript must be enabled to view this page.

members
magnitude
magnitudeUnassigned
count
unassigned
taxon
rank

38945\_out

3

2
3
superkingdom

phylum
3
1239

186801
3
class

3
order
186802

186803
3
family

3
genus
1769710

3
species

SRS019601\_contig\_number\_contig-100\_7060.47416SRS097889\_contig\_number\_contig-100\_984.206839SRS146888\_contig\_number\_contig-100\_3249.102370
1653434
